# Parental Conflicts and Posttraumatic Stress of Children in High-Conflict Divorce Families

**DOI:** 10.1007/s40653-021-00410-9

**Published:** 2021-10-27

**Authors:** Aurelie M. C. Lange, Margreet M. Visser, Ron H. J. Scholte, Catrin Finkenauer

**Affiliations:** 1 Department of Psychiatry, Section of Medical Psychology & Psychotherapy, Erasmus Medical Center, Rotterdam, Netherlands; 2 Viersprong Institute for Studies On Personality Disorders, Halsteren, Netherlands; 3 Children’s Trauma Center, Kenter Youthcare, Amsterdam, Netherlands; 4 Behavioural Science Institute, Radboud University, Nijmegen, Netherlands; 5 Tranzo Tilburg University, Tilburg, Netherlands; 6 Department of Interdisciplinary Social Science: Youth Studies, Utrecht University, Utrecht, Netherlands

**Keywords:** High-conflict divorce, Parental conflict, Interparental contact, Post-traumatic stress, Intervention

## Abstract

Parental conflicts consistently predict negative outcomes for children. Research suggests that children from high-conflict divorces (HCD) may also experience post-traumatic stress symptoms (PTSS), yet little is known about the association between parental conflicts in HCD families and child PTSS. We investigated this association, hypothesizing that parental conflicts would predict child PTSS. We also tested the moderating role of interparental contact frequency, hypothesizing that frequent contact would intensify the association between parental conflicts and child PTSS. This study was part of an observational study on the outcomes of No Kids in the Middle (NKM), a multi-family group intervention for HCD families. A total of 107 children from 68 families participated in the study with at least one parent. We used pre- (T1) and post-intervention (T2) data. Research questions were addressed cross-sectionally, using regression analyses to predict PTSS at T1, and longitudinally, using a correlated change (T1 to T2) model. The cross-sectional findings suggested that mother- and child-reported conflicts, but not father-reported conflicts, were related to the severity of child PTSS. Longitudinally, we found that change in father-reported conflicts, but not change in child- or mother-reported conflicts, were related to change in child PTSS. The estimated associations for the different informants were not significantly different from one another. The frequency of contact between ex-partners did not moderate the relationship between parental conflicts and child PTSS. We conclude that there is a positive association between parental conflicts and child PTSS in HCD families independent of who reports on the conflicts.

**Supplementary Information:**

The online version contains supplementary material available at 10.1007/s40653-021-00410-9.

In the Netherlands, about 86.000 children are involved in a divorce each year (Ter Voet, 2019). Although most parents can handle the aftermath of these divorces reasonably well, 4% to 25% of the divorces involve ongoing bitter conflicts (Fischer et al., 2005; Qu et al., 2014; Smyth & Moloney, 2019). This type of divorces is called a high-conflict divorce (HCD) and is characterised by pervasive negative exchanges between ex-partners in combination with an insecure and hostile emotional environment. Ex-partners are entrenched in pervasive negative interpersonal dynamics, which are characterised by blame, hostility, anger, and fixed negative perceptions of each other (Anderson et al., 2011; Smyth & Moloney, 2019).

Parental conflict consistently predicts negative outcomes for children (Teubert & Pinquart, 2010; van Dijk et al., 2020). Child negative outcomes may be even more pronounced when parental conflicts consist of high levels of hostility or aggression, are child-related (the disputes are about the children), or when the parents involve their children in their conflicts (triangulation), which is most likely to happen in HCD disputes (Hetherington, 2006; McCoy et al., 2013; Van Dijk et al., 2020; Van Eldik et al., 2020). Data from a recent study showed that children involved in high-conflict divorces may be at an increased risk of developing a posttraumatic stress disorder (PTSD), with almost half (46%) of the children being at an increased risk for developing PTSD (van der Wal et al., 2019). This percentage is not far below what has been observed for other types of traumatic experiences in childhood. For example, 67% of children exposed to interparental violence (Georgsson et al., 2011) and 53% of children who were clinically referred after experiencing one or more traumatic events (Verlinden et al., 2014) reported an increased risk for developing PTSD.

The study by van der Wal and colleagues (2019) is the only study, as far as we know, that has investigated the risk of PTSD in children from HCD families. Despite the current relative lack of data to support it, scholars have voiced their concerns regarding the potential impact of HCD on the development of PTSD in children (Davidson et al., 2014). Although research on PTSD in HCD families is lacking, there is a vast body of research on the damaging effect of interparental violence (IPV) on PTSD. A meta-analysis showed that IPV has a large effect on PTSD (Evans et al., 2008) and recent research suggests that IPV can predict PTSD even into young adulthood (Haj-Yahia et al., 2019). IPV occurs when children hear, see, or are directly involved in physical or sexual assaults between their caregivers or parents. In HCD families, children repeatedly hear, see or are involved in verbal conflicts. The first aim of the current study was to examine whether parental conflicts are related to child post-traumatic stress symptoms (PTSS) in this HCD context.

Although IPV can have long-lasting effects on children, there is some evidence that decreases in IPV or parental conflicts may also have a positive effect on the children. Several studies have shown that increases in marital conflict were associated with increasing depressive symptoms and rule-breaking behavior in adolescents, whereas decreasing marital conflicts were associated with decreases of depressive symptoms and rule-breaking behavior (El-Sheikh et al., 2019; Madigan et al., 2017). This latter result is promising and hopeful, as it suggests that interventions focusing on diminishing parental conflicts may have a direct positive impact on child well-being. It is not yet known whether child PTSS might also be related to changes in IPV or parental conflicts.

There is, however, some research on how changes in trauma-eliciting situations might affect PTSD. For example, a study among asylum-seeking children showed that children whose asylum application had been accepted showed fewer post-traumatic stress symptoms at follow-up than children whose application had been rejected (Müller et al., 2019). Although change was not directly tested, their data do suggest that children whose application was accepted experienced a decrease of their PTSS, whereas the other children did not. This study implies that the level of PTSS can be affected by changes in the trauma-eliciting situation and that PTSS might decrease if some aspects of the traumatic event are decreased or are taken away. The current study explored whether similar processes are present in HCD families by not only investigating the relation between parental conflicts and child PTSS concurrently, but also longitudinally.

## Interparental Contact

One potentially important aspect of the trauma-eliciting situation in HCD families is the frequency of interparental contact. Research has shown that ex-partners with children are more likely to persevere in their conflicts than ex-partners without children. The shared responsibility for the children condemns ex-partners to continued interactions over time (Fischer et al., 2005). Indeed, HCD parents have described their interparental encounters concerning child arrangements to be highly stressful to them (Target et al., 2017). Frequent contact may even exacerbate parental conflicts; Kluwer (2016) showed that ex-spouses’ unforgiving emotions were related to higher levels of conflict when the contact frequency was high. It thus may benefit the children if parents can reduce their encounters to a minimum, without necessarily decreasing parent–child encounters, as warm parent–child contacts with both parents may be beneficial for child well-being (e.g., Harper & Fine, 2006; Nielsen, 2017). Unfortunately, little is known about the effect of the frequency of *interparental* contact. We tested whether interparental contact frequency moderates the relationship between parental conflict and child PTSS. We hypothesized that parental conflicts have a stronger impact upon child PTSS when the level of interparental contact is high.

### The Current Study

The current study aimed to fill a gap in the literature by studying intergenerational spillover from parental conflicts to child PTSS in the context of HCD families. The current study only included families without current IPV. Although a previous study on this sample (van der Wal et al.*,* 2019) has found that PTSS is high for children in HCD families, this study extends these findings by investigating the role of conflict; studying whether the severity of and change in child PTSS is related to the severity of and change in parental conflicts. This was done by analyzing whole families (children and both parents) concurrently and longitudinally (over two time points). Moreover, we studied the moderating role of interparental contact frequency. The frequency of contact between ex-partners may exacerbate the effect of conflicts on child PTSS. Research so far has not paid much attention to the frequency of interparental contact.

The current paper is part of a larger multi-wave study on the outcomes of No Kids in the Middle (NKM), a multi-family group intervention aimed at diminishing and de-escalating parental conflicts and developing constructive communication regarding the children (Visser & van Lawick, 2021). The current study had two aims. First, we tested whether parental conflicts predicted child PTSS in a sample of HCD families participating in the intervention NKM. Second, we tested whether this effect was moderated by the frequency of interparental contact. We tested this both concurrently (at the start of the intervention) and longitudinally (studying the change between the start and end of the intervention). This led to the following hypotheses:

- H1: Parental conflicts are related to child PTSS at the start of the intervention.
- H2: Parental conflicts are related more strongly to child PTSS when interparental contact frequency is high than when interparental contact frequency is low (i.e., moderation of contact frequency).
- H3: Change in parental conflicts between the start and end of the intervention is related to change in child PTSS.
- H4: Change in parental conflicts between the start and end of the intervention is more strongly related to change in child PTSS when interparental contact frequency is high than when interparental contact frequency is low (i.e., moderation of contact frequency).

Parental conflicts were measured from the perspective of both parents, as well as from the perspective of the child.

## Methods

### Participants and Procedure

Families participating in the NKM intervention between April 2014 and March 2016 were asked to participate in the study. All families were referred by judges or child protection services (CPS) to a health care institution because the wellbeing of the children was threatened by the ongoing parental conflict. A total of 302 parents were asked for their participation of whom 203 (67%) signed the informed consent. Of these 203 families, 24 did not participate in the research and another twelve decided not to start or to discontinue the intervention, resulting in a sample of 167 parents participating in the study (55% of the 302 parents who were approached). There were 56 couples and 55 parents participated alone. Only parents whose children participated in this study, were included in the analyses for this manuscript. Assessments took place at the start (T1) and (T2) end of the intervention, which was approximately 4 months apart.

Children between 6 and 18 years old could participate in the research if both of the legal parents signed for consent. Children of 12 years and older also had to sign the assent form themselves. In total, 193 children were invited to participate, of which 144 children received and gave permission (75%). As the questionnaire regarding PTSS was designed for children of 8 years and older, younger children were excluded for this particular manuscript (*n* = 28). For another 9 children, none of the parents provided data in the study. Therefore, the final sample consisted of 107 children between 8 and 18 years old in 68 families. All children participated with at least one parent. The sample consisted of 35 families with one participating child, 27 families with 2 participating children and 6 families with 3 participating children. Of the 68 families, 49 families had both parents participating in the study, meaning that most children (70%, *n* = 75) were participating in the study with both of their parents. Families took part in the intervention across 14 different institutions in 24 different intervention groups.

### Intervention

No Kids in the Middle (NKM) consists of eight parallel parental and children group sessions of maximal six parental couples (over a period of approximately four months). To be admitted, both parents need to participate in the intervention. At the same time, children groups take place, consisting of all children aged 4 to 18 years that are involved in the HCD divorces of the parents in the intervention. The children groups primarily focus on support and empowerment. Research shows that parents participating in NKM reported decreased parental conflicts up to six months post-intervention (Lange et al. submitted; Visser et al. 2020). The presence of IPV was an exclusion criteria, meaning that none of the families had ongoing IPV during the study.

### Measures

#### **PTSS**

Children’s traumatic impact of the high-conflict divorce of their parents was measured with the Children’s Revised Impact of Event Scale (CRIES-13; originally developed by Horowitz et al., 1979; translated to Dutch by Van der Ploeg et al., 2004), which provides a stable assessment of traumatic impact across different types of trauma and life threatening events (e.g., Perrin et al., 2005) and has been found to be reliable and valid (Verlinden et al., 2014). Children rated 13 items assessing the frequency of the occurrence of these events in the past week in relation to the conflicts and divorce of their parents (1 = *never*, 2 = *rarely*, 3 = *sometimes*, and 4 = *often*). Example items are: “Do you think about the divorce and conflicts of your parents even when you don’t mean to?”, and “Do you avoid talking about the divorce and the conflicts of your parents?” We used the sum score as an indicator of traumatic impact (ranging from 0 to 65). A score of 30 and greater has been suggested as the most efficient cut-off for discriminating heightened risk for PTSD (Verlinden et al., 2014). In our sample, 46% of the children had a heightened risk for PTSD at T1 and 34% at T2. Reliability of the scale was good based on Guttman’s lower bound (*λ*_2_ = 0.87 at T1 and *λ*_2_ = 0.90 at T2; Guttman, 1945).

#### **Conflict, Parent-reported**

As co-parenting conflict (i.e., the degree to which parents agree or disagree about child-related issues) is one of the most devastating types of conflicts for children (Van Eldik et al., 2020), we specifically focused on co-parenting conflicts for the parent-reported conflicts. Parental perception of co-parenting conflict was measured using the co-parenting conflict scale from the Psychological Adjustment to Separation Test (PAST; Sweeper & Halford, 2006; translation by De Smet et al., 2012). The scale consisted of seven items on a 5-point scale (1 = *totally disagree*, 5 = *totally agree*). Example items are “My former partner and I arrange child visitation well” (reversed) and “I fight with my former partner over the well-being of the child/children”. Higher average scores (range = 1–5) indicate higher levels of co-parenting conflict. Father-reported and mother-reported conflicts were used independently in the analyses to allow for studying all family members’ perspectives. Reliability of the scale was good based on Guttman’s lower bound (*λ*_2_ varied between 0.75 and 0.84 over time for men and women).

#### **Conflict, Child-reported**

Children reported to what extent their parents fought in their presence (1 item) on a 5-point scale (1 = *never*, 2 = *sometimes*, 3 = *regularly*, 4 = *often*, and 5 = *always*).

#### **Frequency of Contact**

Frequency of contact was assessed through three questions completed by the parents, namely ‘How often do you talk to your ex-partner personally?’, ‘How often do you talk to your ex-partner through the phone?’ and ‘How often do you have written contact with your ex-partner?’ All three questions could be answered on a five-point scale (1 = *(almost) never*, 2 = *less than once a month*, 3 = *once a month*, 4 = *several times a month*, 5 = *more than once a week*). Clinical experience of the second author indicated that children might not only be stressed when their parents actually meet one another personally, but also be stressed by interparental phone and text contact, for example if the sound of incoming text messages is always followed by anger and distress of the parent. We therefore decided to take all these different types of interparental contact into account by calculating the average of these three questions. We did not analyse parents separately, but rather used an average of mothers and fathers as the closest representation of the actual contact frequency between parents. A higher score (range = 1–5) indicated a higher frequency of contact (independent of the medium). Reliability of the scale was good based on Guttman’s lower bound (*λ*_2_ = 0.86 at T1 and *λ*_2_ = 0.87 at T2).

### Analytical Plan

#### **Missing Data and Nesting of Data**

We experienced non-response at both time-points, with different rates of non-response for the different informants. Non-response at the start of treatment was 1% for the children and 15% for fathers and 15% for mothers. Non-response at the end of treatment was 16% for children, 41% for fathers and 32% for mothers. All children had data of at least one parent at the start and 85% had data of at least one parent at the end of treatment. There were no missing data for age and gender of the child. For the remaining variables, available data ranged between 77 and 85% at T1, and between 55 and 77% at T2. A quarter of the families (27%, *n* = 29) had complete data (no missing data on any of the assessments for any of the family members). These families did not differ on any of the variables from families with partially missing data, except for PTSS at the start of the intervention, which was lower for families with complete data compared to families with partially missing data (*t* (88) = 2.60, *p* < 0.05). To account for missing data, all data was imputed 40 times using Bayesian estimation in Mplus, using an unrestricted (H1) variance–covariance two-level model (children nested in families) (Asparouhov & Muthén, 2010; Graham et al., 2007).

All analyses were conducted in Mplus 8 (Muthén & Muthén, 1998–2017). We accounted for the non-independence of families by adjusting the standard-errors using the COMPLEX module in Mplus. For conflict, separate analyses were run for each informant (father, mother and child), as we were interested in these different perspectives. For contact frequency, the average of both informants (fathers and mothers) was used to calculate the amount of contact between parents at that time-point as an estimation of the actual contact frequency between parents. Prior to all analyses, we checked the bivariate correlation matrix. Age and gender of the child were included as covariates in subsequent analyses if they were significantly correlated (*p* < 0.05) to one of the included variables.

#### **Effect of Conflict on PTSS at Start of the Intervention (H1) and Moderator Effect (H2)**

We tested conflict as predictor of PTSS using three hierarchical linear regression analyses (as we had three conflict informants). We added contact frequency in the second step and an interaction term between contact frequency and conflict in the third step. The interaction term was included to test for moderation of contact frequency (H2). All included variables were assessed at T1. As these models were saturated, they had a perfect fit and model fit was not reported.

#### **Correlated Change (H3) and Moderator Effect (H4)**

**Fig. 1:**
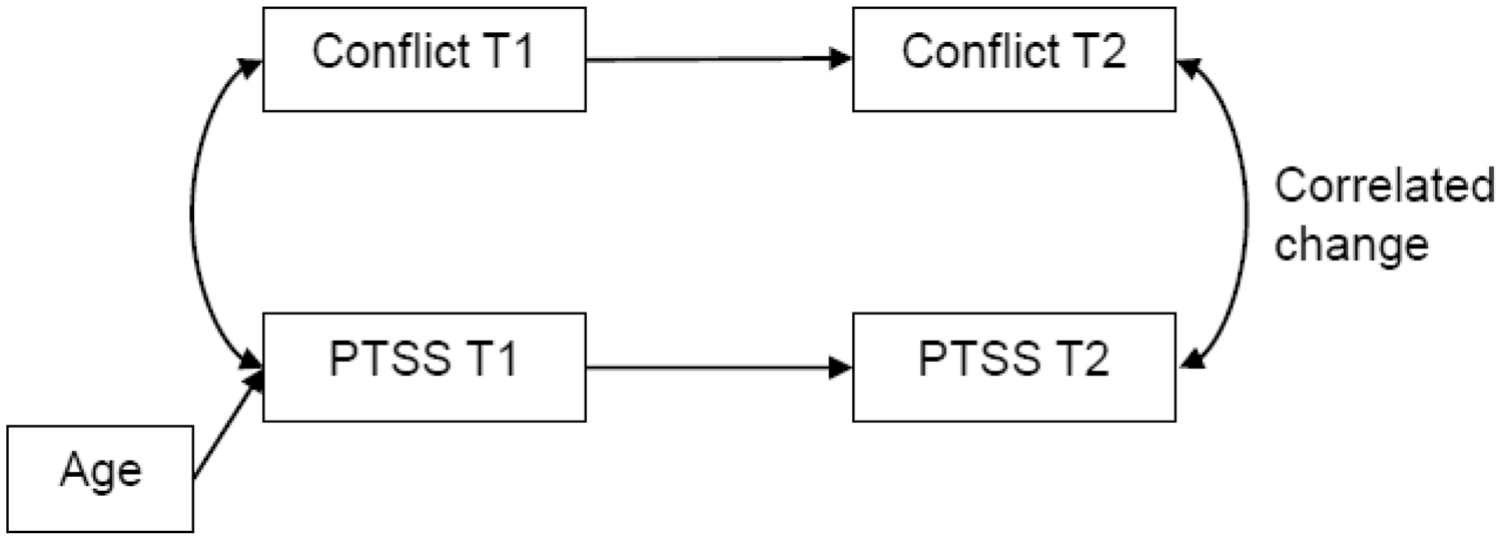
Diagram of analytical model, representing the correlated change between conflict and PTSS between T1 and T2

We tested whether change in conflict and change in PTSS during the intervention were related to one another. Although we planned to use correlated latent change models, model fit for these models was poor. We therefore choose to use a path model whereby T2 of both conflict and PTSS were regressed on T1 of the same variable. We also included an association between both variables at T1 and T2. The association at T2 can be interpreted as correlated change (Asendorpf & van Aken, 2003). Figure 1 provides a graphical representation of the tested model.

We subsequently tested whether frequency of contact was a moderator using multi-group analyses. For this purpose, we split the families into two groups: one group that reported an increase over time of the intervention in contact frequency (59%) and one group that reported a decrease over time in contact frequency (41%). Using the Satorra and Bentler (2001) scaled chi-squared difference test, we tested an unrestricted model against a model in which all parameter estimates were restricted to be equal across these two contact groups. As the number of families in each group varied across the imputed datasets, it was not possible to conduct these moderator analyses on the imputed data. Therefore, the moderator analyses were conducted on the original non-imputed data. The *n* used in these analyses varied between 76 and 88 (71% to 82% of the total sample).

#### **Sensitivity Analyses**

Although all families were referred because of the ongoing parental conflicts, some families had only recently separated. Divorcing couples are likely to have more severe conflicts just after separation due to the stress of the new situation, but these may not be enduring for multiple years, as is the case in HCD families (Smyth & Moloney, 2019). We therefore replicated all analyses for the subset of families that had separated for more than two years (*n* = 89) to exclude families that potentially had situational rather than persisting parental conflicts. We only reported the results of these sensitivity analyses if they differed from the original analyses on the full sample.

## Results

Table 1 presents descriptive statistics for all variables. Bivariate correlations are presented in Table 2. There was a moderate correlation between fathers’ and mothers’ conflict reports, however only weak and sometimes non-significant correlations between children and parents. Interestingly, contact frequency was negatively related to the level of conflict according to parents, but not related to the level of conflict according to the children. Thus, parents reporting higher levels of conflict tended to report lower levels of contact and vice versa. This association was significantly stronger for mothers than for fathers at T2 but not at T1. The amount of conflict reported by children, however, was not related to the frequency of contact between parents. Child’s age was correlated to their level of PTSS and was therefore included as covariate in all analyses. Gender was only significantly related to the level of co-parenting conflict according to the mother and was therefore only included as a covariate in analyses regarding mother-reported conflicts.

**Table 1:** Descriptive statistics for imputed data (N = 107)

	T1	T2
	**%**	**%**
Gender child: boy	55%	
Do your parents fight in front of you?		
Never	39%	55%
Rarely	48%	32%
Regularly	2%	6%
Often	5%	5%
Always	5%	1%
	***M* (sd)**	***M* (sd)**
Age child	11.12 (2.79)	
Age father	44.48 (7.34)	
Age mother	42.05 (5.07)	
Duration of relationship (years)	13.10 (5.48)	
Duration of separation (years)	5.19 (2.48)	
PTSD symptoms (0–65)	27.37 (15.83)	24.48 (17.48)
Co-parenting conflict father (1–5)	3.50 (0.72)	3.26 (0. 83)
Co-parenting conflict mother (1–5)	3.26 (0.72)	3.16 (0.72)
Level of contact father (1–5)	2.68 (1.14)	2.71 (1.24)
Level of contact mother (1–5)	2.58 (1.14)	2.83 (1.14)

**Table 2:** Pearson’s correlation for imputed data

	1	2	3	4	5	6	7	8	9	10	11	12	13	14
1. Conflict T1 Father	-													
2. Conflict T1 Mother	.52***	-												
3. Conflict T1 child	.16	.28**	-											
4. Conflict T2 Father	.65***	.43***	.28**	-										
5. Conflict T2 Mother	.52***	.77***	.14	.56***	-									
6. Conflict T2 child	.22*	.22*	.41***	.33***	.18	-								
7. PTSS T1	.09	.28**	.22*	.08	.25**	.04	-							
8. PTSS T2	.17	.11	.14	.33***	.16	.19*	.54***	-						
9. Contact T1 Father	–.28**	–.20*	–.13	–.16	–.12	.05	–.14	–.08	-					
10. Contact T1 Mother	–.28**	–.49***	–.11	–.06	–.41***	.04	–.13	.04	.69***	-				
11. Contact T2 Father	–.27**	–.12	–.17	–.31**	–.13	.03	–.12	–.13	.77***	.45***	-			
12. Contact T2 Mother	–.27**	–.37***	–.01	–.36***	–.61***	.08	–.07	–.07	.48***	.52***	.56***	-		
13. Age child	–.00	.04	.07	.07	.04	.12	–.19*	–.10	–.17	–.18	–.10	–.06	-	
14. Gender child	–.13	–.19*	.02	–.08	–.17	.05	.12	.06	–.01	.02	–.02	.02	–.13	-

^*^p < .05; **p < .01; ***p < .001

### Effect of Conflict on PTSS at Start of the Intervention (H1) and Moderator Effect (H2)

We first tested hierarchical linear regressions with child PTSS at T1 as the dependent variable (see Table 3). The level of parental conflicts, as perceived by the mother and the child predicted PTSS; more conflicts were related to higher PTSS. Father-reported parental conflicts were not related to PTSS. The estimates for the three informants were not significantly different from one another. When adding contact frequency, mother- and child-reported conflict continued to predict child PTSS. Contact frequency did not predict child PTSS, nor did the interaction between contact frequency and conflict predict child PTSS. Thus, our results suggest that contact frequency did not moderate the association between conflict and child PTSS.

**Table 3:** Hierarchical regression analyses of child PTSS at T1

	Regression 1	Regression 2	Regression 3
	B (s.e.)	B (s.e.)	B (s.e.)
	Mother-reported conflict		
Age child	–1.16 (0.7)	–1.25 (0.72)	–1.25 (0.71)
Gender child	5.00 (3.06)	4.81 (3.10)	4.82 (3.09)
Conflict	6.57 (2.13)**	6.00 (2.45)*	3.28 (5.22)
Contact		–1.14 (1.75)	–4.09 (4.89)
Contact * Conflict			0.94 (1.65)
*R*^*2*^	.15*	.16*	.16*
	Father-reported conflict		
Age child	–1.22 (0.72)	–1.45 (0.78)	–1.43 (0.78)
Conflict	1.88 (2.61)	0.65 (2.55)	–3.37 (5.45)
Contact		–2.73 (1.69)	–7.54 (4.78)
Contact * Conflict			1.43 (1.46)
*R*^*2*^	.05	.08	.08
	Child-reported conflict		
Age child	–1.32 (0.71)	–1.52 (0.78)	–1.54 (0.78)*
Conflict	3.44 (1.41)*	3.16 (1.37)*	4.79 (3.56)
Contact		–2.49 (1.60)	–1.28 (3.05)
Contact * Conflict			–0.67 (1.37)
*R*^*2*^	.09	.12	.12

^*^p < .05; **p < .01

In the sensitivity analyses, only including families that had separated for more than two years, the level of conflicts according to children was no longer a significant predictor of child PTSS (*B* (s.e.) = 2.80 (1.50), *p* = 0.06).

### Correlated Change (H3) and Moderator Effect (H4)

Secondly, we tested the correlated change between parental conflict and child PTSS. For all models, model fit was excellent (see Table 4). For father-reported conflict, change in conflict was positively related to change in PTSS, indicating that decreasing levels of conflict were related to decreasing levels of child PTSS (B (s.e.) = 2.84 (1.06), *p* = 0.01). Change in child- and mother-reported conflict was not significantly associated with change in child PTSS (child-reported conflict: B (s.e.) = 2.40 (1.35), *p* = 0.08; mother-reported conflict: B (s.e.) = 0.65 (0.87), *p* = 0.45). Again, however, the estimates for the three informant groups were not significantly different from one another.

**Table 4:** Model fit indices for path models representing correlated change

	Mean model fit indices for imputed data					Model fit indices for original data				
*Model*	χ^2^	CFI	TLI	RMSEA	SRMR	χ^2^	CFI	TLI	RMSEA	SRMR
Father-reported conflict	5.43	0.99	1.00	0.03	0.05	7.05	0.96	0.93	0.06	0.07
Child-reported conflict	7.65	1.00	1.06	0.00	0.06	1.95	1.00	1.14	0.00	0.04
Mother-reported conflict	4.52	1.00	1.04	0.00	0.04	1.62	1.00	1.11	0.00	0.03

Before conducting the multi-group analyses on the original non-imputed data to test for moderation of interparental contact frequency, we replicated the path models on the original data. The findings replicated the results of the imputed datasets regarding the effect of parental conflict on child PTSS (father-reported conflict: B (s.e.) = 4.42 (1.23), *p* = 0.000; child-reported conflict: B (s.e.) = 2.52 (1.26), *p* = 0.05; mother-reported conflict: B (s.e.) = 1.22 (0.77), *p* = 0.11). When conducting the multi-group analyses on the original data, we found that the restricted model, in which all parameters were restricted to be equal across the two groups representing increasing or decreasing levels of contact, was not significantly different from the unrestricted model in all analyses. This means that contact frequency was not a significant moderator.

## Discussion

The aim of this study was twofold. First, we tested the association between parental conflicts and child PTSS, concurrently and longitudinally, assuming that parental conflicts would be positively associated to child PTSS. Concurrently, we found that both mother- and child-reported conflicts, but not father-reported conflicts, were related to the severity of the PTSS. Longitudinally (between the start and end of the intervention NKM), change in father-reported, but not mother- or child-reported conflict, was related to change in child PTSS. Secondly, we examined the moderating role of interparental contact frequency, hypothesizing that more frequent contact might intensify the association between conflict and PTSS. This hypothesis was not confirmed; interparental contact frequency did not moderate the association between conflict and PTSS.

The current study showed a robust association between parental conflicts and child PTSS in HCD families, as these findings were found for multiple informants, were replicated in most of the sensitivity analyses, and were found using two different analytical approaches. As far as we know, this is the first time that child PTSS has directly been related to parental conflicts in HCD families. Since IPV was an exclusion criteria for participation in the intervention, this study provides evidence that verbal conflicts, without IPV, may also be associated to child PTSS. It is important for practitioners to be attentive to PTSS in children of divorcing couples, especially when parental conflicts are severe. Although parental conflicts may not be the only factor of a divorce that can be stressful for children (e.g., moving houses, losing contact with friends or family), the ongoing and severe conflicts are likely to be an important aspect of the divorce trauma in HCD families. It is known that prolonged traumatic stress or accumulation of traumatic events can increase the risk of PTSD (e.g. Müller et al., 2019), yet less is known about the link between decreases in the trauma-eliciting event and subsequent changes in PTSS. In this study, conflicts decreased on average, suggesting that decreases in parental conflicts may be related to decreases in PTSS. Moreover, as parental conflicts, but not child PTSS, were directly targeted by the intervention, these results cautiously suggest that decreases in parental conflict can lead to decreases in child PTSS. This adds to the preliminary available evidence that taking away or diminishing part of the trauma-eliciting event can have a direct positive impact on PTSS. We must, however, bear in mind that this was a correlational study, and hence, causation cannot be tested. It is equally likely that changes in child PTSS led to changes in parental conflicts. For example, parents in HCD tend to fight for the benefit and the good of their children (Anderson et al., 2011; Target et al., 2017). Perceiving PTSS in their child might be a reason for parents to intensify conflicts with their ex-partner, assuming that their ex-partner is the cause of their child’s distress.

Although the results were somewhat different for different informants (i.e., mother-reported and child-reported conflict were related to child PTSS at the start of the intervention, whereas father-reported *change* in conflict was related to *change* in child PTSS), the results did not significantly differ between informants, suggesting that, overall, father-, mother- and child-reported conflict had a similar relation to child PTSS. Previous research has found that conflicts may have a more severe impact on children through fathers than mothers (Cummings et al., 2010). Parenting behavior is an important mediator through which parental conflicts affect children (van Dijk et al., 2020). Several studies suggest that the spillover effect from parental conflicts to parenting behaviors, such as parental distress or parent–child hostility, is stronger for fathers than for mothers (Camisasca et al., 2016; Cummings et al., 2010; Harold et al., 2013). According to the fathering vulnerability hypothesis, fathers might be more vulnerable to experience marriage-related disruption in their parenting than mothers because the distinction between their roles of husband and father might be less distinct (Cummings et al., 2010). Not all studies, however, find support for this notion of vulnerability; studies have reported that parents either have similar effects on children or that mothers and fathers affect children differently through different mechanisms (e.g., Nelson et al., 2009; Ponnet et al., 2013). The current study adds to this body of evidence, suggesting that both parents have equal effects on their children.

Child-reported conflict was no longer a significant predictor of child PTSS in the sensitivity analysis, suggesting that child-reported conflict might be a less robust predictor of child PTSS in families with enduring and long-lasting conflicts. The child-reported conflicts were assessed with only a single item, assessing the frequency with which parents had conflicts in front of them. In HCD families with enduring conflicts, the actual frequency of conflicts in the child’s presence might be less important than the destructivity of the conflicts, or how the conflicts affect the children through other processes such as parenting behaviors.

Contrary to our hypothesis, we did not find a moderating effect of interparental contact frequency. Although unexpected, this finding is consistent with a related study on interparental contact frequency and child wellbeing after divorce. Kluwer (2016) showed that parental revenge motivations in divorced parents were positively associated with parental conflicts, which, in turn, were negatively associated with child well-being. This association was not moderated by interparental contact frequency. Although Kluwer (2016) studied child well-being rather than child PTSS, she used the same measure to assess interparental contact frequency. Thus, research so far seems to suggest that interparental contact may not intensify the association between conflicts and child outcomes. This does, however, not mean that interparental contact is of no relevance for parental conflicts or child outcomes. Reducing contact with the ex-partner may be a tactic parents use to reduce their conflicts with their ex-partner. NKM therapist may also encourage ex-couples to reduce their interparental contact frequency if reducing the conflicts through other means seems especially hard.

Indeed, the bivariate correlations of this study showed that frequent interparental contact was related to lower rather than higher frequencies of conflict. This suggests that parents reporting many conflicts may try to avoid contact with their ex-partner. It is interesting that this effect was stronger for mothers than fathers. This could be because of the more ‘secure’ position of mothers, as they are more frequently the primary caregiver of the children after divorce (Kalmijn, 2015). This was also the case in our study, where 40% of the children lived primarily with their mother compared to only 6% of the children living primarily with their father. As such, mothers can more easily decrease contact with their ex-partner without this having any consequences for the frequency with which they see their children. Fathers, on the other hand, may need to ‘fight’ for the right to see their children (Target et al., 2017), which means they might be more inclined to keep in touch with their ex-partners. Over time, conflicts seemed to decrease, whereas contact frequency slightly increased, suggesting that ex-partners may also increase their contact if their interactions improve over time.

This study has several limitations. First, we only had data for two time points, relatively close in time to one another (four months). Replicating and extending our findings in longitudinal studies with more time points would be a promising avenue. Longitudinal observational studies could investigate how parental conflict and child PTSS co-develop and mutually influence one another over time. Alternatively, an RCT of an intervention addressing parental conflicts would allow to test causality and study whether an intervention can decrease child PTSS by reducing parental conflicts. A second limitation is the use of self-reports instead of observation of conflicts. Parents’ perception of the conflicts may not align with actual levels of parental conflicts, which may blur the relationship between parental conflicts and child PTSS. This is exemplified by the moderate correlation between fathers’ and mothers’ perception of their mutual conflicts. Nevertheless, our findings were not significantly different between informants, suggesting that parental conflicts are related to child PTSS independent of who reports on the conflicts. Lastly, although our focus was on conflicts, many other aspects of a divorce may be stressful for children, such as moving houses, court involvement, reduced income of the parents, or the sudden loss of their secure family as a basis (Amato, 2010). Also child maltreatment or interparental violence may play a role in HCD families and impact upon child PTSS (Beck et al., 2013). Future research into child PTSS in the context of HCD needs to pay attention to these other aspects to develop a more comprehensive understanding of the experience of HCD for children and the role of the parental conflicts within this context.

It is important to note we had to use a different analytical approach than originally planned for our analyses of correlated change due to poor model fit. Although the path model used may be less intuitive for the analysis of correlated change than the latent change model, both the path model (Asendorpf & van Aken, 2003) and the latent change model (Könen & Auerswald, 2021) are appropriate for assessing correlated change and produced very similar estimates. We therefore do not feel this shift in analytical approach has impacted upon the trustworthiness of the results.

This study also has several strengths. First, we used a multi-informant and systemic approach, investigating parental conflicts through the eyes of both parents, as well as the children involved, and looking at an intergenerational spillover effect. Second, we included a large age-range (8–18 years) and an equal number of boys and girls in the study, and we controlled for age and gender in the analyses, allowing these results to be generalizable to a large group of children in HCD families. Third, we managed to include the fathers of 85% of the children in this study. Although fathers’ behavior and characteristics are related to children’s normal and abnormal development, fathers are underrepresented in child psychopathology research (Parent et al., 2018), as well as in therapeutic treatment of children’s mental health (Tully et al., 2017). The current study found some differential results for fathers and mothers, supporting the necessity of including and studying the role of fathers in children’s mental health. Last, this study was novel in its analyses of interparental contact frequency. For this purpose, we used an inclusive measure, assessing all types of contact, namely face-to-face, phone and written contact.

### Clinical Implications and Conclusions

The high level of PTSS observed in this sample of children from HCD families underlines that professionals working with these families need to be sensitive to potential child PTSS. Incorporating a trauma narrative into an intervention can strengthen children processing the traumatizing events they may have been exposed to by growing up in HCD families (Cohen et al., 2012). The current study further suggests that professionals need to carefully observe the relationship between the interparental contact frequency and the parental conflicts within each family. Although reducing contact may be a strategy to reduce conflict, we found that low contact was actually related to high levels of conflict. As causality could not be tested, we do not know whether parents with frequent conflicts avoid one another, or whether avoidant parents get into more conflicts due to a lack of communication. Lastly, NKM seems to be a promising intervention; preliminary evidence suggests it can decrease parental conflicts and may, through that, decrease child PTSS. We do, however, must bear in mind that this multi-wave study did not include a control group, meaning that more research is needed into the effectiveness of NKM and similar programs targeting parental conflicts in HCD families.

To conclude, this study addressed a gap in our understanding of the intergenerational spillover of HCD by examining how parental conflicts related to child PTSS, concurrently and longitudinally. It not only demonstrated that parental conflicts in families experiencing a high-conflict divorce are related to child PTSS, but also that changes in parental conflicts may be related to changes in child PTSS. This study highlights that child PTSS is an important theme within HCD families and that parental conflicts may play a significant role into its development. We look forward to future research investigating how PTSS is affected by different aspects of the divorce and the behaviors of the parents in HCD families. As such, we can build towards more evidence-based interventions to protect and support children and help parents realize positive change.

## Supplementary Information

Below is the link to the electronic supplementary material.

Supplementary file1 (DOCX 12 KB)
