# Supplementary material for: Parental Conflicts and Posttraumatic Stress of Children in High-Conflict Divorce Families
Source: J Child Adolesc Trauma. 2021 Oct 27;15(3):615–25. doi: 10.1007/s40653-021-00410-9 (PMC9360253; doi:10.1007/s40653-021-00410-9)
Supplement: Supplementary file 1 — Supplementary file1 (DOCX 12 KB) [file 40653_2021_410_MOESM1_ESM.docx]

**Supplementary materials**

**Syntax Mplus**

*Regression analysis, including interaction effect*

TITLE: regression analysis;

DATA:

FILE IS implist.dat;

TYPE = imputation;

VARIABLE: conflict1 conflict2 ptss1 ptss2 familyid age;

USEVARIABLES ARE

conflict1 conflict2 ptss1 ptss2 age contact1 int;

MISSING ARE all(999);

CLUSTER = familyid;

DEFINE:

int = contact1*conflict1;

ANALYSIS:

TYPE = complex;

MODEL:

ptss1 on age1 conflict1 contact1 int;

*Path analysis*

TITLE: path model;

DATA:

FILE IS implist.dat;

TYPE = imputation;

VARIABLE: conflict1 conflict2 ptss1 ptss2 familyid age;

USEVARIABLES ARE

conflict1 conflict2 ptss1 ptss2 age;

MISSING ARE all(999);

CLUSTER = familyid;

ANALYSIS:

TYPE = complex;

MODEL:

ptss1 with conflict1;

ptss2 with conflict2;

conflict2 on conflict1;

ptss2 on ptss1;

ptss1 on age;

*Path analysis with multigroup analysis (restricted)*

TITLE: path model;

DATA:

FILE IS originaldata.dat;

FORMAT = 6f8.2;

VARIABLE: conflict1 conflict2 ptss1 ptss2 familyid age group;

USEVARIABLES ARE

conflict1 conflict2 ptss1 ptss2 age;

MISSING ARE all(999);

CLUSTER = familyid;

GROUPING = group (0 = less 1 = more);

ANALYSIS:

TYPE = complex;

MODEL:

ptss1 with conflict1;

ptss2 with conflict2;

conflict2 on conflict1;

ptss2 on ptss1;

ptss1 on age;

MODEL LESS:

ptss1 with conflict1 (1);

ptss2 with conflict2 (2);

conflict2 on conflict1 (3);

ptss2 on ptss1 (4);

ptss1 on age (5);

MODEL MORE:

ptss1 with conflict1 (1);

ptss2 with conflict2 (2);

conflict2 on conflict1 (3);

ptss2 on ptss1 (4);

ptss1 on age (5);
